# Over Six Thousand *Trypanosoma cruzi* Strains Classified into Discrete Typing Units (DTUs): Attempt at an Inventory

**DOI:** 10.1371/journal.pntd.0004792

**Published:** 2016-08-29

**Authors:** Simone Frédérique Brenière, Etienne Waleckx, Christian Barnabé

**Affiliations:** 1 IRD-CIRAD, INTERTRYP (Interactions hôtes-vecteurs-parasites-environnement dans les maladies tropicales négligées dues aux Trypanosomatidés), IRD Center, Montpellier, France; 2 Pontificia Universidad Católica del Ecuador, Centro de Investigación para la Salud en América Latina (CISeAL), Quito, Ecuador; 3 Centro de Investigaciones Regionales “Hideyo Noguchi”, Universidad Autónoma de Yucatán, Mérida, Yucatán, México; US Food and Drug Administration, UNITED STATES

## Abstract

*Trypanosoma cruzi*, the causative agent of Chagas disease, presents wide genetic diversity. Currently, six discrete typing units (DTUs), named TcI to TcVI, and a seventh one called TcBat are used for strain typing. Beyond the debate concerning this classification, this systematic review has attempted to provide an inventory by compiling the results of 137 articles that have used it. A total of 6,343 DTU identifications were analyzed according to the geographical and host origins. Ninety-one percent of the data available is linked to South America. This sample, although not free of potential bias, nevertheless provides today’s picture of *T*. *cruzi* genetic diversity that is closest to reality. DTUs were genotyped from 158 species, including 42 vector species. Remarkably, TcI predominated in the overall sample (around 60%), in both sylvatic and domestic cycles. This DTU known to present a high genetic diversity, is very widely distributed geographically, compatible with a long-term evolution. The marsupial is thought to be its most ancestral host and the Gran Chaco region the place of its putative origin. TcII was rarely sampled (9.6%), absent, or extremely rare in North and Central America, and more frequently identified in domestic cycles than in sylvatic cycles. It has a low genetic diversity and has probably found refuge in some mammal species. It is thought to originate in the south-Amazon area. TcIII and TcIV were also rarely sampled. They showed substantial genetic diversity and are thought to be composed of possible polyphyletic subgroups. Even if they are mostly associated with sylvatic transmission cycles, a total of 150 human infections with these DTUs have been reported. TcV and TcVI are clearly associated with domestic transmission cycles. Less than 10% of these DTUs were identified together in sylvatic hosts. They are thought to originate in the Gran Chaco region, where they are predominant and where putative parents exist (TcII and TcIII). Trends in host-DTU specificities exist, but generally it seems that the complexity of the cycles and the participation of numerous vectors and mammal hosts in a shared area, maintains DTU diversity.

## Author Summary

*Trypanosoma cruzi*, the causative agent of Chagas disease, has been classified into six genetic groups (discrete typing units, DTUs) named TcI-TcVI and a seventh one called TcBat. Currently, several genetic molecular markers are used to classify the strains after their isolation in culture or directly from biological samples. The current inventory compiling the published works aiming to identify the DTUs of *T*. *cruzi* strains accumulated a total of 6,343 identifications. Although this inventory is not free of sampling bias, like all samples, it is the largest sampling to date and hence likely represents the closest picture of the current diversity of *T*. *cruzi* strains (i) circulating throughout the endemic area from the southern United States to Argentina and (ii) circulating in vectors as well as in wild and domestic mammals, and humans. Data analysis helps identify trends and provides a basis for further comparisons of new data, in a context where human factors (migration, vector control, urbanization, deforestation, agricultural expansion, resource exploitation) influence the epidemiological patterns of Chagas disease.

## Introduction

*Trypanosoma cruzi* is a pathogenic microorganism, the causative agent of Chagas disease, characterized by high genetic and phenotypic intraspecific diversity. Population genetics suggests that clonality is an important mode of propagation of the natural populations of *T*. *cruzi* [1], although, likely sexual reproduction [2, 3] and recombination events occur to some extent and are important mechanisms that generate genetic diversity within the taxon, as discussed in a recent review [4].

The consensual nomenclature recognizes six discrete typing units (DTUs) named TcI to TcVI and a recently proposed seventh, Tcbat [5–7]. This classification is widely used as a reference in epidemiological studies. However, there is not consensus on the best method to identify the different DTUs. Similarly, the evolutionary relationships between the DTUs and therefore the evolutionary history of *T*. *cruzi* continue to be researched [8]. Several mechanisms of evolution have been recognized such as clonality, hybridization, and conventional and nonconventional genetic exchanges. In addition, several studies have demonstrated the extraordinary plasticity of the *T*. *cruzi* genome. The evolutive relationships among these DTUs has not been fully elucidated, but two of them (TcV and TcVI) clearly have a hybrid origin with TcII and TcIII as putative parents [9] according to the authors, TcIII and TcIV could also originate from a hybrid between TcI and TcII [10, 11] but some claim that is not the case [12, 13]. TcI and TcII remain two pure lines that are evolving separately from a common ancestor dating from approximately 1–3 million years ago [11, 13].

The main properties of the different DTUs have been reported previously [3, 5, 14, 15]. Briefly, (i) TcI has a wide distribution, from the southern United States to northern Argentina and Chile; this DTU is the most frequently sampled in sylvatic cycles, but it is also frequent in domestic cycles and it is the dominant DTU responsible for the transmission of Chagas disease in endemic countries located north of the Amazon basin; (ii) studies show that TcII, V and VI are more likely to be associated with domestic cycles and patients with chronic Chagas disease in the Southern Cone countries and Bolivia; (iii) TcIII and IV are mainly sampled in rainforest sylvatic cycles; (iv) Tcbat previously identified in bats, has recently been found in humans [7, 16–18]. It is well known that various DTUs can coexist in the same vector and in a single host [19–21].

The different DTUs present substantial genetic diversity. Various reports have shown that the parasite’s genetic diversity has a profound impact on its epidemiological, biological and medical characteristics [22]. Consequently, it is indispensable to characterize the genotypes that are circulating in space and in hosts. Moreover, the tracking of the different genotypes is of great interest in eco-epidemiology, providing a better understanding of epidemiological systems.

After the biogeographic overview of *T*. *cruzi* DTUs by Miles and his colleagues [23], no other exhaustive review has been done, while very numerous new genotyping studies using new genetic markers and additional parasite strains have been conducted. Although we are conducting studies on the limits of DTUs classifications of *T*. *cruzi* strains and their actual existence as genetically separated units, it seemed important to take all existing data that refer to the current classification and to examine the geographic properties and host specificities of the different DTUs.

## Methods

Data were obtained from a total of 137 articles (including our own published results) selected after searching PubMed (http://www.ncbi.nlm.nih.gov/pubmed) with “DTU”, “genetic characterization”, “lineage”, “genotype”, “isozyme”, “isoenzyme”, and “*Trypanosoma cruzi”* as key words. This research, as exhaustive as possible, was updated to April 27, 2016. Research has also been conducted by authors having worked on the genetic characterization of *T*. *cruzi* strains. For our published data, additional data, not present in the publications, was included in the current inventory because this information was available from our own records. For example, the names and data concerning the strain origins analyzed in Barnabé et al. [24] were added here. The publications included in the inventory used genetic markers that allowed DTU typing according to the consensual nomenclature in 6–7 DTUs [5, 6]. Moreover, in some cases correspondences between typing methods with different markers were used for the data interpretation [6, 25]. The data are shown in an Excel spreadsheet (S1 Table) where each line corresponds to a single determination from an isolate, a strain, a laboratory clone, mammal blood or tissue samples, and different vector digestive tract samples (“sample type” column in S1 Table). Several lines were recorded when different DTUs were detected in a strain and its laboratory clones. When more than 1 DTU was detected in one vector or mammal host (mixed infection), several lines corresponding to each DTU were recorded in the file. A total of 6,343 determinations were compiled. Each of them has a code corresponding to the strain/sample name reported in the publications, except for the records not identified with a name but only counted in publications, which we have labeled “anonymous”. In a few publications, undistinguished DTUs were reported for part of the identifications; consequently, additional categories were created for them: TcI/TcII (three cases), TcII/TcV/TcVI (26 cases), TcII/TcVI (two cases), TcIII/TcIV (31 cases), and TcV/VI (47 cases). These undistinguished DTUs accounted for 1.7% of the total inventory. The geographical origin was informed by the country name (no missing data), the upper continental subdivision of North, Central, and South America, the upper administrative divisions such as state, province, department or region, and the lower administrative divisions such as municipality, province, or community according to the information existing in the publications. The collection dates of the strain or biological samples were not always documented (52.5% of missing year data). Host origin was generally informed by the species (31 missing data), and columns were added indicating the order, genus and tribe for the triatomines. Also, the cycles to which the different hosts belonged were classified as “domestic” when the hosts were living and/or were captured in the intra- and peridomicile areas, and “sylvatic” when the hosts were captured in the field outside domestic areas. When the location of the capture site was missing, the wild mammals where classified in the sylvatic cycle except for the synanthropic species such as opossums and rodents for which the information was considered as unknown (uk). The information on the methods used for the characterization of the DTUs is also included in S1 Table. The first column indicates if the DTU was characterized at nuclear or mitochondrial level or both, the second one indicates the method(s) used, and the third one on the markers, the names of the genes, or the number of loci for MLMT (multilocus microsatellite typing) and MLEE (multilocus enzyme electrophoresis).

## Results

### General overview of available data

The 6,343 samples of *T*. *cruzi* DTUs compiled in this review were identified in vectors and mammalian hosts from 19 different countries, covering an area from the southern United States to Argentina (S2 Table). No data is available from Belize in Central America, and Uruguay and Guyana in South America. The vast majority of data relate to South America (90.7%). The DTUs were identified in 86 genera (32 missing cases), 158 different species of which 42 are vectors belonging to 7 genera (*Dipetalogaster*, *Eratyrus*, *Meccus*, *Mepraia*, *Panstrongylus*, *Rhodnius*, and *Triatoma*). Approximately of the identifications in South America 49.3% were from vector species; however, in North and Central America most of the identifications were from vectors (69.3% and 65.8% respectively). The mammal species belong to nine orders of which the most represented is the Primate order (61.5%), because 59.4% of the identifications in mammals were made in samples from humans (n = 1902). One-third of the DTU identifications (31.0%) corresponded to parasites from hosts (vectors and mammals) captured in sylvatic ecotopes, 57.6% from intra- and peridomestic hosts, and the others were undetermined (n = 719, 11.3%) because in several studies the origin of the vectors was not specified.

### Overall distribution of the DTUs (TcI-TcVI and Tcbat)

**Fig 1:**
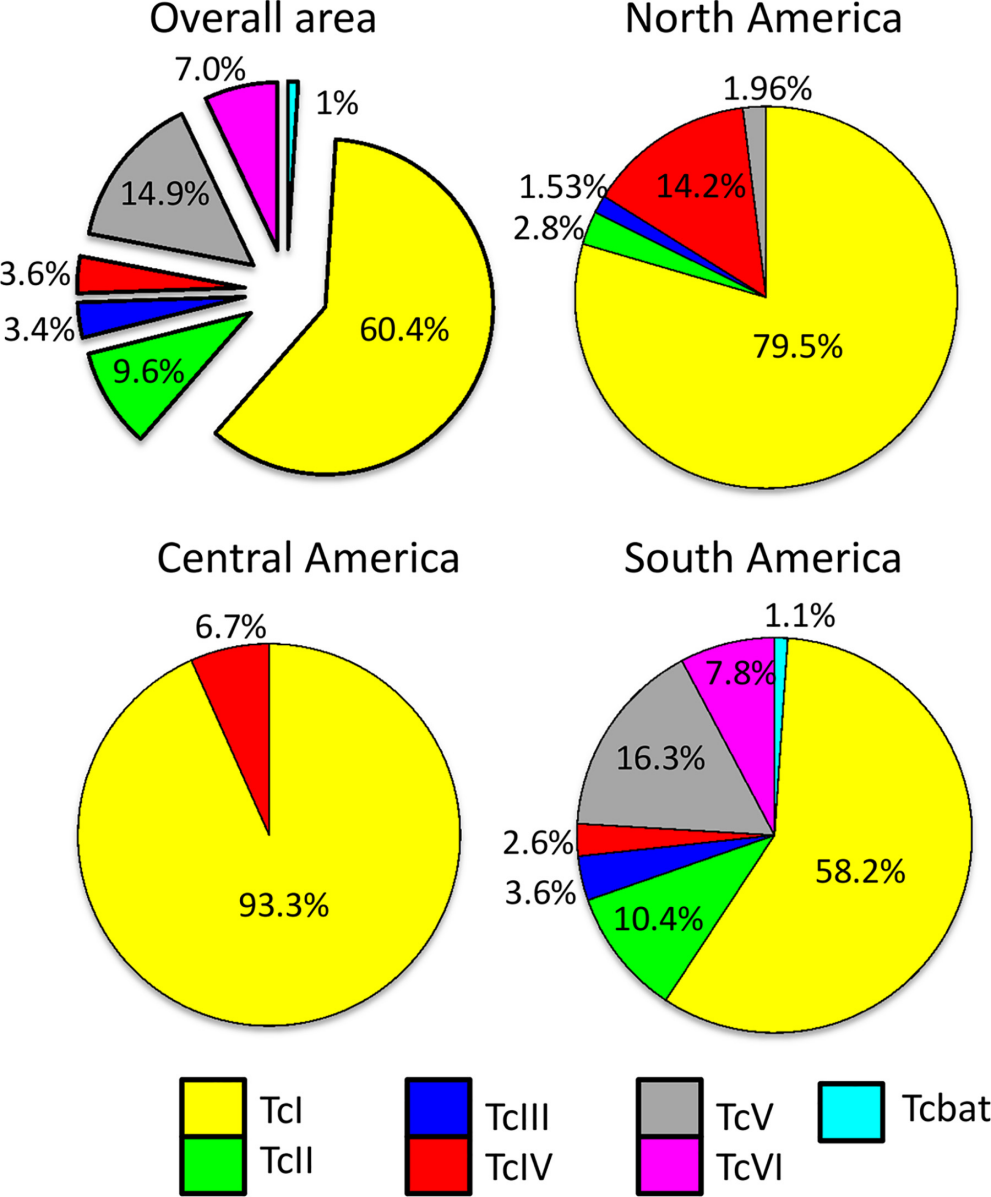
*T*. *cruzi* DTUs distribution (TcI-TcVI and Tcbat) over 6234 determinations in vector and mammalian hosts from 19 endemic countries in the overall endemic area: In North America (n = 459), Central America (n = 120) and South America (n = 5655). The ambiguous determinations of DTUs were deleted from the samples.

In 1.7% of the samples, the DTU (n = 109) was reported as a group of DTUs: (i) in one dog, 15 coati from Brazil, and ten triatomines from Argentina, TcII, TcV, and TcVI were not distinguished; (ii) TcII or TcVI was reported in two *T*. *infestans* from Paraguay; (iii) 47 infections with TcV or TcVI in dogs, humans, *T*. *infestans* from Chile and Bolivia and *P*. *megistus* in Brazil were reported; (iv) in 31 vectors and mammal hosts from Brazil and Mexico TcIII/TcIV were not discriminated; and (v) in three cases TcI and TcII were not discriminated in *T*. *pallidipennis*. In the 6234 other records, TcI was found in approximatively 60.0% of the overall identifications; TcII, TcV and TcVI were identified in around 10% each; and TcIII, TcIV and Tcbat were rarer with percentages ≤ 3.6%. Fig 1 presents the proportions of DTUs observed, excluding from the calculation the ambiguous DTU determinations over the entire endemic area, and in North, Central and South America (see below).

### Geographical distribution of the DTUs

**Fig 2:**
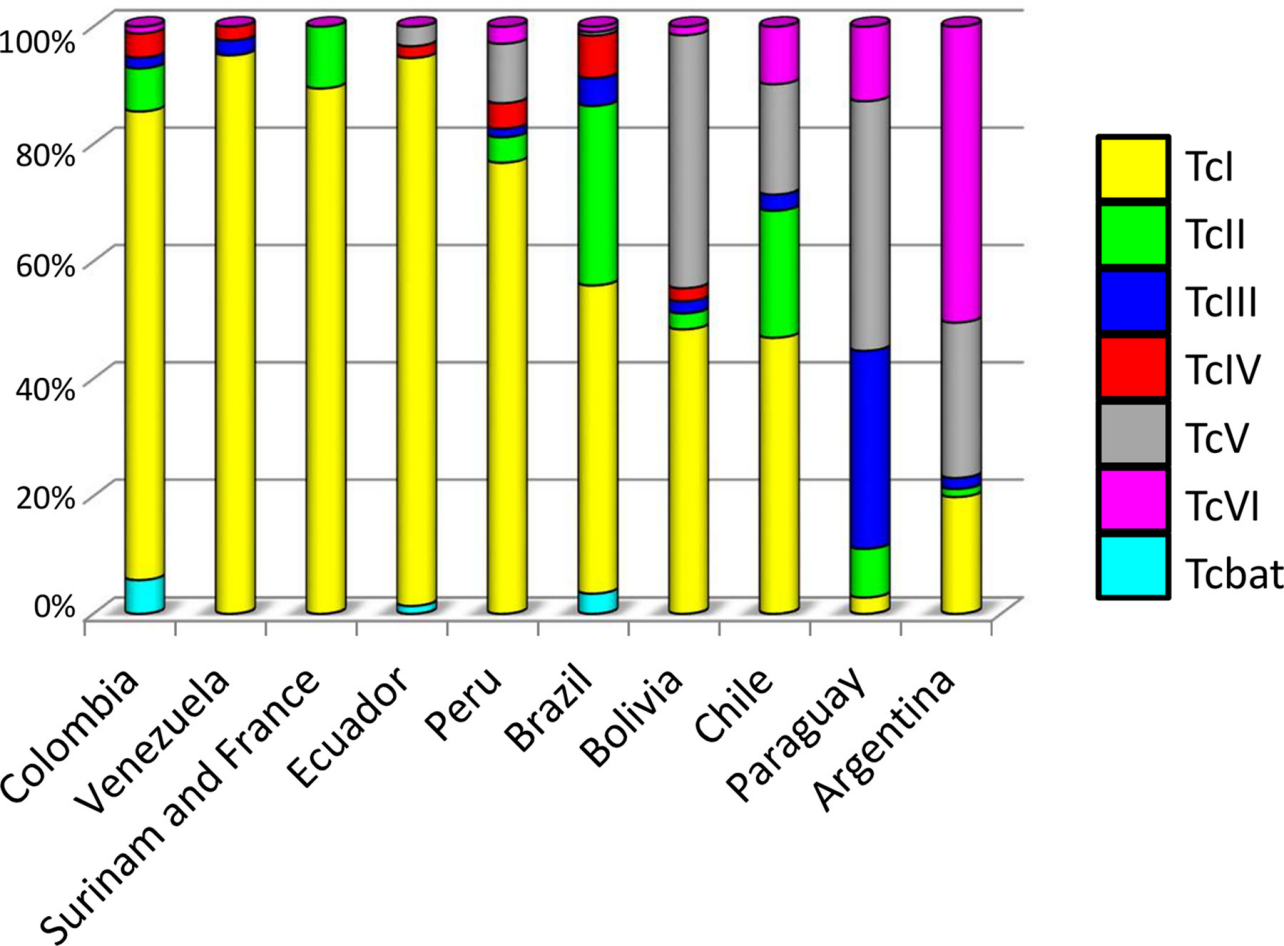
*T*. *cruzi* DTU distribution per country in South America out of a total of 5655 identifications. The ambiguous determinations of DTUs were deleted from the samples.

According to the current available records, the DTU distribution was different between North, Central, and South America (Fig 1). In Central America only two DTUs (TcI and TcIV) were identified while all DTUs were detected in South America. In North America the latest studies have identified TcII, TcV and TcIII in addition to TcI and TcIV, which remain the major strains, in Central America. In South America the DTU distribution was highly variable depending on the country, and the current trend is a predominance of TcI north of the Amazon and the presence of all DTUs south of the Amazon with abundance of TcV and TcVI (Fig 2).

Tcbat is a recently proposed DTU that is genetically more closely related to TcI than to any other DTU. Therefore this DTU is probably underestimated because it is not recognized by the markers used in many publications, and consequently it may have been erroneously equated with TcI. This DTU was identified in 59 bats belonging to 12 different species in Brazil, Colombia, and Ecuador [16, 17, 26, 27], in one specimen of *T*. *sordida* from the State of Mato Grosso do Sul State in Brazil [28], and in a Colombian patient infected with a mixture of TcI and TcBat [18].

As mentioned above, TcI was the most frequently identified DTU in the overall sample, with a lower percentage in South America (58.2%) than in North America (79.5%) and Central America (93.3%). It was identified in all the countries included in the study. In South America, the low frequencies of TcI in Argentina (19.9% of 589 determinations) and Paraguay (2.8% of 181) contrasted with the proportions of this DTU in the other South American countries (at least > 47.0%) (Fig 2).

TcII was much more rarely identified (9.6% of overall DTUs identified). It was not identified in Central America out of 120 identifications, and only 13 identifications were reported from North America out of 459 (2.8%). Eight of these 13 TcII were found in Mexico, four in *T*. *dimidiata* captured in domestic cycles in the state of Veracruz [29] and four in *Meccus pallidipennis* collected in Michoacan [30]. The five other identifications were from mice and rats captured in the immediate surroundings of the dwelling of the first described autochthonous case of *T*. *cruzi* transmission in Louisiana, near New Orleans [31, 32]. In South America, TcII presents a higher proportion, reaching 10.4% and was reported in Colombia, Surinam, Peru, Bolivia, Brazil, Argentina, Paraguay and Chile.

TcIII and TcIV, which are thought to result from ancestral hybridization between TcI and TcII, reached 3.4% and 3.6% of the identifications, respectively. In North America, both of these DTUs were reported in Mexico in several publications [29, 30, 33, 34], but for the moment only TcIV has been identified in the US [24, 31, 35, 36]. In Central America, only TcIV has been identified in Guatemala in humans and vectors [37, 38]. In other Central American countries, neither TcIII nor TcIV has been reported. In South America, TcIII could be more cosmopolitan (Argentina, Bolivia, Brazil, Chile, Colombia, Paraguay, Peru and Venezuela) than TcIV, which has not yet been identified yet in Argentina, Chile and Paraguay.

The last two DTUs, TcV and TcVI, were the recent hybrids, derived from hybridizations between TcII and TcIII. These DTUs showed the most differential geographical distribution. Indeed, TcV was identified in North America in exceptional cases in Mexico (Veracruz) in *T*. *dimidiata* as well as above-mentioned TcII [29]. TcV and TcVI have never been identified in US in 148 determinations, nor in Central America in 120 cases. In contrast, in South America, these DTUs together have frequently been identified in several countries, Argentina (76.9%), Bolivia (44.6%), Chile (28.6%) and Paraguay (55.2%)—but very rarely in others such as in Colombia (1.1%) [24, 39–41], Ecuador (3.3%) [42], and Brazil (1.5%) [24]. In Peru they were identified in 13.0% [24, 43, 44]. Moreover, when the two DTUs coexist, different proportions can be observed in the different countries. The most remarkable case was the identification of TcV and TcVI in Bolivia with 43.1% and 1.0% respectively, while in Argentina TcVI was more common (50.0%) and TcV less frequently detected (26.5%).

### Eco-epidemiology of the DTUs

#### Domestic versus sylvatic cycles

**Fig 3:**
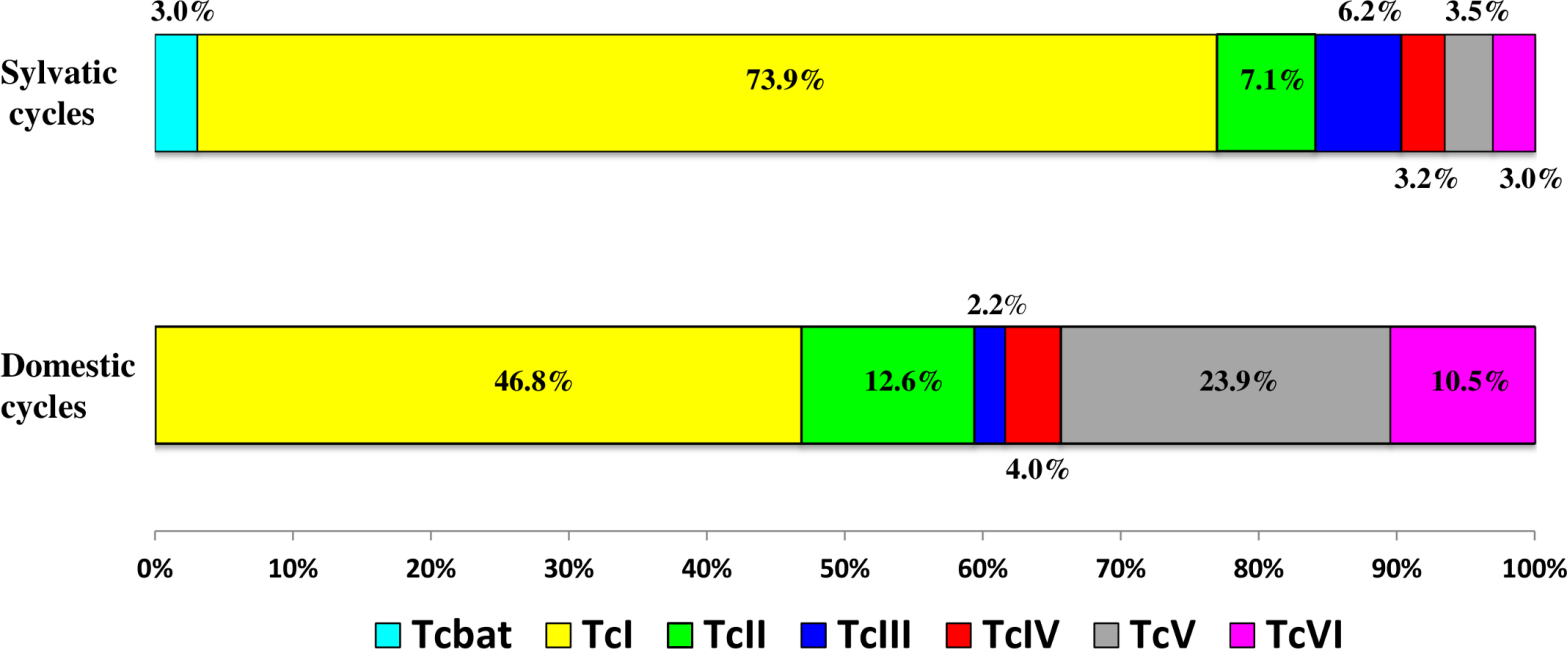
Differential *T*. *cruzi* DTUs distributions in wild and domestic cycles. The ambiguous determinations of DTUs were deleted from the samples.

*T*. *cruzi* circulates in nature in different environments and two categories are usually distinguished: (i) the domestic cycles where *T*. *cruzi* evolves between domestic vectors, domestic and synanthropic mammals, and humans that are living in dwellings or/and around dwellings in the peridomestic areas; see Walter et al. for a comprehensive definition of peridomicile [45]; and (ii) the sylvatic cycles where *T*. *cruzi* evolves between wild mammals and vectors living outside domestic areas. The current results (Fig 3) show that all DTUs, including Tcbat and taking into account the two cases described in the domestic cycle [18, 28], participate in domestic and sylvatic cycles in some places. According to the current inventory, TcBat, TcI and TcIII are significantly more frequently identified in sylvatic cycles than in domestic cycles (X^2^ test, *p* < 10^−4^) and inversely for TcII, TcIV TcV and TcVI (X^2^, *p* < 10^−4^). Nevertheless, these tests are only indicative because they correspond to a very gross approach that ignores sampling bias, which obviously exists.

#### Vector infections

To date, TcI is the major DTU identified in vectors (78.1%). TcI was also the only DTU identified in the genus *Eratyrus* (n = 6) or was very predominant (> 91%) in *Meccus* (n = 176), *Panstrongylus* (n = 715), and *Rhodnius* (n = 525) (Table 1).

**Table 1:** DTUs of *T*. *cruzi* currently detected in seven genera of *T*. *cruzi* vectors.

Vector genus	DTU of *T*. *cruzi*							
	TcBat	TcI	TcII	TcIII	TcIV	TcV	TcVI	Total
*Dipetalogaster*		1						1
*Eratyrus*		6						6
*Meccus*		161	4	2	9			176
*Mepraia*		113	52			29	26	220
*Panstrongylus*		689	5	20	1			715
*Rhodnius*		499	3	1	21		1	525
*Triatoma*	1	952	48	43	12	180	200	1436
Total	1	2421	112	66	43	209	227	3079

For these genera where TcI was found highly prevalent, it is useful to detail which are the other DTUs identified: (i) in the genus *Meccus* TcII, TcIII, and TcIV were identified in one report in the species *M*. *pallidipennis* collected in municipalities of the State of Michoacan de Ocampo in Mexico [30]. In this study, of 26 specimens of this species, TcI only reached 42.3%; TcIII or TcIV have also been detected in *T*. *longipennis* in Jalisco state [34]. (ii) In the genus *Panstrongylus*, besides TcI, the dominant DTU (96.7%), TcII was identified in Brazil, TcIII in sylvatic cycles in three countries (Brazil, Colombia and Venezuela) and in domestic cycles in Bolivia [46], and TcIV [47] and TcV or TcVI were identified once in Venezuela and Brazil respectively [13, 47]. The hybrid strains (TcV or TcVI) were identified in *P*. *megistus* collected in Minas Gerais (Brazil) in a domestic environment. These results suggest a remarkably high diversity of DTUs in this genus. (iii) In the genus *Rhodnius*, besides TcI, the other DTUs were very scarce. Among them, TcIV was the most common (4.0%). It was identified in three species: *R*. *brethesi* and *R*. *robustus* in the Brazilian Amazon and *R*. *prolixus* in Colombia [24, 39], Venezuela [47] and Guatemala [37]. TcII was identified in only three *R*. *neglectus* and *R*. *pictipes* bugs in the state of Para in Brazil [48, 49]. Finally, TcIII and TcVI were reported in a single individual each (*R*. *brethesi* and *R*. *prolixus* respectively) [39, 50].

In contrast, in the genera *Mepraia* and *Triatoma* although TcI remains a major strain (51.4% and 66.3% respectively), the other DTUs were found more frequently.

In *Mepraia*, the identifications were made in two species (*M*. *gajardoi* and *M*. *spinolai*) captured in a sylvatic environment for which, in addition to TcI, remarkably high percentages of TcII (23.6%), TcV (13.2%) and TcVI (11.8%) were identified [51–53].

In the genus *Triatoma*, the data were available for 18 species (Table 2), but the results concerned principally *T*. *infestans* (1081 identifications, 73.8%). Lower numbers of DTU identifications were available for *T*. *dimidiata* (170), *T*. *sordida* (50), *T*. *barberi* (46), T. rubida (24) *T*. *maculata* (19), *T*. *eratyrusiformis* (14) and *T*. *protracta* and *T*. *braziliensis* (12). For the remaining species, there were fewer than ten identifications.

**Table 2:** DTUs of *T*. *cruzi* currently detected in the genus *Triatoma*.

Species	DTU of *T*. *cruzi*							
	Tcbat	TcI	TcII	TcIII	TcIV	TcV	TcVI	Total
*Triatoma barberi*		46						46
*Triatoma brasiliensis*		6	3					9
*Triatoma carrioni*		3						3
*Triatoma dimidiata*		143	4	5	9	9		170
*Triatoma eratyrusiformis*		6					1	7
*Triatoma gerstaeckeri*		7						7
*Triatoma infestans*		627	37	31	1	170	194	1060
*Triatoma maculata*		19						19
*Triatoma matogrossensis*			1					1
*Triatoma nigromaculata*		3						3
*Triatoma nitida*		1						1
*Triatoma protracta*		11			1			12
*Triatoma pseudomaculata*		4						4
*Triatoma rubida*		24						24
*Triatoma rubrovaria*				7				7
*Triatoma sanguisuga*		8			1			9
*Triatoma sordida*	1	40	3			1	5	50
*Triatoma venosa*		2						2
Total	1	950	48	43	12	180	200	1434

In this set of species, TcI was very dominant (>80%) except in *T*. *infestans* and *T*. *braziliensis* where it was less abundant (59.1% and 66.6%respectively). In *T*. *infestans* all DTUs were identified. TcI, TcV and TcVI dominated (93.5%), while TcII and TcIII accounted for about 6.4% together and TcIV was only detected once in an endemic valley in southern Peru. Although *T*. *brasiliensis* has an epidemiological importance in Brazil, few strains were identified in this vector, all from states located in the Northeast region in Brazil (N = 12) mostly from domestic cycles: six TcI, three TcII, and three TcIII or TcIV.

In the other species where TcI predominated, other DTUs were identified. In *T*. *dimidiata*, TcI was the only DTU identified except in one study in which of 33 specimens of the Mexican state of Veracruz, TcI (nine cases) as well as TcII, TcIII, TcIV and TcV were identified, the latter accounting for 72.7% of the sample. In *T*. *sordida*, TcII was detected in Brazil [28, 54], and TcV and TcVI in Argentina [55], and most of the insects were captured in domestic cycle.

For the following nine species with a sample size < 10 (*T*. *carrioni*, *T*. *gerstaeckeri*, *T*. *matogrossensis*, *T*. *nigromaculata*, *T*. *nitida*, *T*. *pseudomaculata*, *T*. *rubrovaria*, *T*. *sanguisuga*, and *T*. *venosa*), TcI was a major DTU (28/37, 75.7%), TcII was identified in one *T*. *matogrossensis* [28], TcIII was the only DTU identified in *T*. *rubrovaria* from one study in the State of Rio Grande do Sul in Brazil [7], and TcIV was identified in one *T*. *sanguisuga* in the US. The hybrid DTUs (TcV and TcVI) were not identified [24].

#### Mammal infections

**Fig 4:**
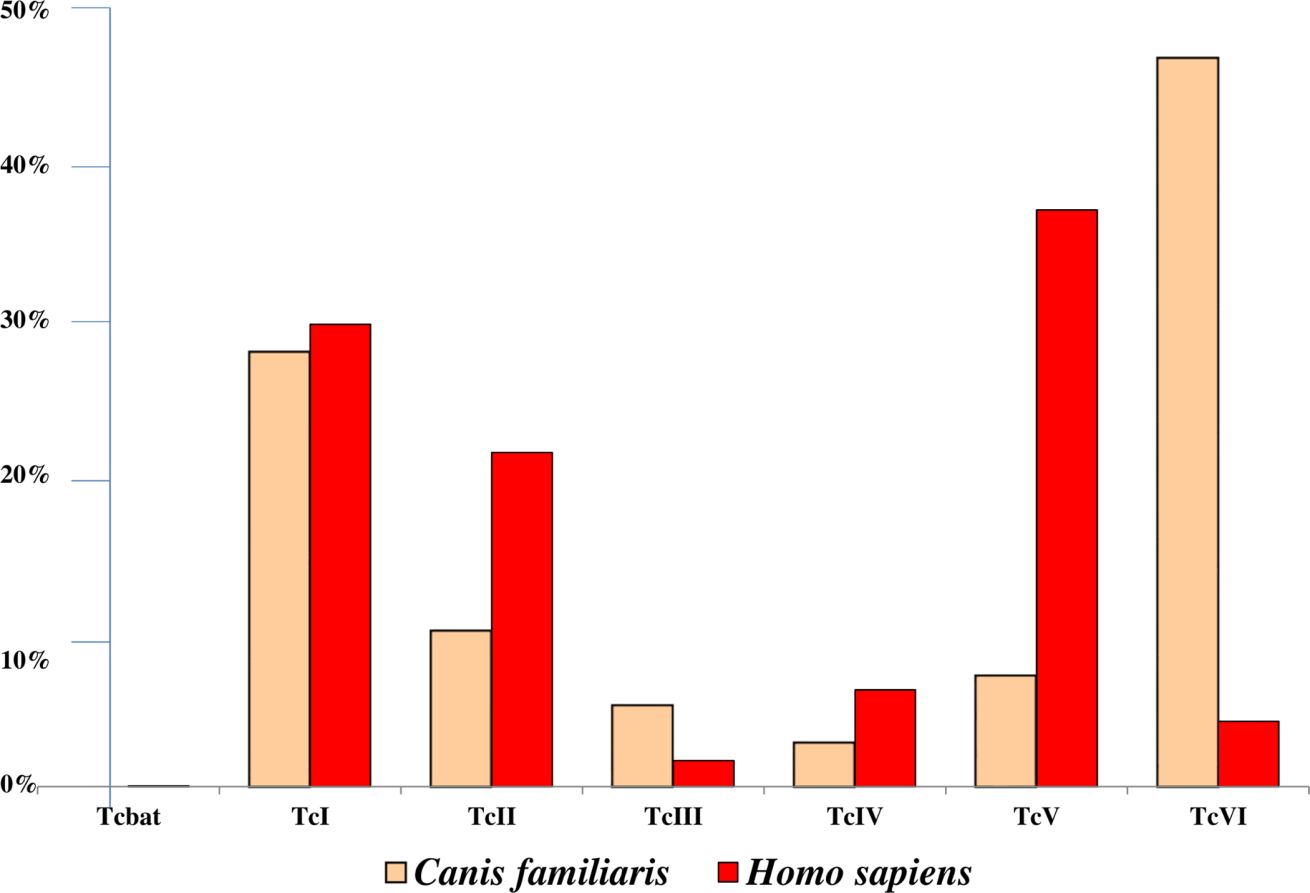
Comparative distribution of the *T*. *cruzi* DTUs in humans and dogs.

*Domestic cycles*. Considering domestic cycles and their mammalian hosts, most of the identifications made were from samples isolated from humans (1902 identifications, 84.0%). Those in dogs reached 220, from nine countries. Sixteen identifications were from cats from Argentina [56, 57], 91 others were from small rodents in six countries that readily nest in peridomestic structures (e.g., in sheds and piles of building materials), including 52 reports in *Rattus rattus* from Venezuela. There was also a single study of DTU identification in 24 goats in Chile [58]. In North America and Central America together (n = 112), there were 76 identifications in humans, accounting for 4.0% of the total number of human identifications, ten in dogs, and 26 in small rodents; mostly TcI was found (85.7%). The other DTUs were TcII in small rodents from the US [31] and TcIV from three human cases in Guatemala [37] and in the US two rodents [31] and six dogs [35]. In South America, all DTUs except Tcbat were identified in humans and dogs, and their distribution was rather similar, except for the hybrid DTUs TcV and TcVI (Fig 4). TcV was more abundant than TcVI in humans and conversely in dogs. In both cases, the DTUs TcI, and the hybrid DTUs TcV or TcVI were the most frequently identified, reaching at least 25% each. For goats in Chile, TcI and hybrid strains were found to be abundant, but TcII also reached 29.2% [58]. For small rodents, TcI predominated. However, TcII and TcIV were, together with TcI, recently identified in the peridomestic area of a house surrounded by forest in Louisiana [31]. Moreover, TcV was found in *R*. *rattus* caught in peridomiciles of dwellings in the region of Chiquitania in Bolivia where *T*. *sordida* is the main vector species [59].

*Sylvatic cycles*. In the wild environment, the identifications of *T*. *cruzi* strains isolated from mammals involved nine orders and 106 species for which very few characterizations were available for each one. Currently, TcI appears to be the most frequent DTU (58.3%) in the overall samples. For the orders, Artiodactyla [47, 60] and Pilosa [10, 24, 61, 62], which included no more than five identifications each, all were TcI. For the order Xernathra the two strains identified were TcIII or TcIV [63] (Table 3). For the other orders, several specific trends are detailed below.

**Table 3:** Inventory of DTUs of *T*. *cruzi* identified in 960 wild mammals belonging to nine orders.

Mammal orders	DTU of *T*. *cruzi*									
	Tcbat	TcI	TcII	TcII/TcV/TcVI	TcIII	TcIII/TcIV	TcIV	TcV	TcVI	Total
Artiodactyla		3								3
Carnivora		46	1	15	4	8	36			110
Chiroptera	59	57	21		4		4			145
Cingulata		2	1		78		1	1		83
Didelphimorphia		262	1		7	3		3	2	278
Pilosa		5								5
Primate		43	10		1		10			64
Rodentia		91	37		10	1		24	20	183
Xernathra						2				2
Total	59	509	71	15	104	14	51	28	22	873

Examining the DTU distribution in the main orders and species (Table 3), it is worth noting that TcI reached 94.2% in the order Didelphimorphia, while TcIII reached a similar percentage in Cingulata (94.0%). The 278 identifications in Didelphimorphia were from 12 countries in North and South America, and from 20 species. In this order, only a few of the other DTUs were identified: TcII in Chile [58], TcIII in Brazil [7, 63, 64] and Paraguay [65], TcIII or TcIV in Mexico [34] and TcV and TcVI in Bolivia [24, 59] and Chile [58]. Species from the order Cingulata were sampled in six countries (Bolivia, Brazil, Colombia, Paraguay, United States, and Venezuela) where TcIII was the main DTU, and in the US out of three samples, two were TcIV and one TcI. Two other identifications in Paraguay were TcII and TcV.

In the order Chiroptera, 145 identifications were reported in 23 species, all from South America. Tcbat and TcI were similarly identified (around 40% each), while TcII identified in Brazil, Colombia and Surinam was less frequent (14.5%), and TcIII and TcIV were very rare (2.7% each) [66, 67].

In the order Carnivora, TcI (37.1%) and TcIV (58.1%) were principally identified, but of the 36 TcIV samples, 35 were from *Procyon lotor* captured in the US, also infected with TcI (two cases) and one *Nasua nasua* from Brazil, both belonging to the same Procyonidae family. Four TcIII were identified in *Conepatus chinga* in Argentina. In the order Rodentia, the DTU distribution was quite different with high percentages of TcII (20.0%), TcIII (5.5%), TcV (13.1%) and TcVI (10.1%) in addition to TcI (49.7%). However, the majority of the sample was from Chile (61.7%) and Brazil (27.9%); TcV and TcVI were abundant only in Chile and not in Brazil where TcI was 78.4% and TcIII 17.6%. For the wild primates, a total of 51 identifications were made in 15 monkey species mostly sampled in Brazil (82.8%); TcI (67.2%), TcII (15.6%), TcIV (15.6%) and TcIII in one specimen were the four DTUs identified, TcI (Brazil, Colombia, Ecuador, France and Venezuela) and TcIV (Bolivia, Brazil, USA and Venezuela) were from different countries, while TcII was sampled only in Brazil.

## Discussion

For many years, the characterization of *T*. *cruzi* strains was mostly conducted with specific goals in limited geographical areas and consequently with a limited number of strains. The current compilation, based on the consensus nomenclature of six DTUs, reached an accumulated number of 6,343 identifications. However, *T*. *cruzi* genotyping is associated with many biases and trapping methods, and several caveats must be considered, such as (i) unequal distributions of the research groups in the eco-epidemiology of *T*. *cruzi* in different countries, resulting in nonhomogeneous information; (ii) selection of some DTUs during the culture step; (iii) differential parasitemia levels in hosts, facilitating the isolation by hemoculture or xenodiagnosis, or facilitating the direct detection of some DTUs over others; (iv) markers’ differing ability to detect the different DTUs; (v) overrepresentation of humans in the overall sample; (vi) scarcity of mammals that are difficult to trap; (vi) difficulties discriminating closely related DTUs; and (vii) use of a nonstandardized set of reference strains. Despite of this nonexhaustive list of biases, the data reported herein constitute the most complete picture of the DTU distribution in the endemic area of Chagas disease.

The purpose of this review is not to discuss the current nomenclature of *T*. *cruzi* in six DTUs. Indeed, there is an increasing number of new genetic analyses of *T*. *cruzi* strains, especially from sylvatic cycles, which show that it is increasingly difficult to obtain a relevant genetic structure that divides into six statistically supported clusters with the most in vogue genetic markers, microsatellites and nuclear sequence polymorphisms [68–70]. Moreover, at the mitochondrial level, we recently assessed that three robust clusters that we named mtTcI, mtTcII and mtTcIII actually exist [8]. The mtTcI cluster includes only strains belonging to the TcI DTU, the mtTcII includes only those belonging to the TcII DTU and mtTcIII includes strains belonging to several DTUs: TcIII and TcIV (ancient hybrids of TcI/TcII), TcV and TcVI (recent hybrids TcII/TcIII) and even TcI (a result of mitochondrial introgression for some strains labeled TcI with nuclear markers). These last few years, a number of studies aiming to characterize *T*. *cruzi* strains have used the nomenclature of six DTUs, so we proposed to examine the eco-epidemiological features of these DTUs and highlight new knowledge that may challenge the current paradigm.

### Geographical distribution and origin of the DTUs

Based on the available typing data, the first outstanding result is the predominance of TcI strains. This DTU, genetically diversified, is found throughout the geographic distribution of *T*. *cruzi* and in all cycles where it is always dominant. There are probably no ecological systems (i.e. geographical areas where the parasite evolves between mammalian hosts and vectors specific species) where TcI is absent. However, it appears that TcI strains do not develop well in some mammal species such as those within the order Cingulata since this order is rarely infected with TcI (Table 3). The ecological systems are usually complex networks of relationships involving many species of mammals and vectors, and strain diversity may be maintained because of differential interactions between the parasite’s hosts and genotypes. TcI is an old DTU that has evolved since 3–16 MYA as previously proposed [71], and its very high genetic diversity is consistent with a long-term evolution. Moreover, recombination between TcI strains appears to be more frequent than previously thought [2, 3, 72]. The recombination events (i.e. sex) generally increase the variability of the organisms and thus increase their resilience, allowing new areas to be conquered and especially new hosts that have probably played a key role in the large dispersion and adaptation of TcI. Another question is the geographical origin of TcI. A North-South clustering was recognized, even if some incongruence remains to be explained [73–75]. In an analysis of TcI, the Gran Chaco region was proposed as an origin, while human TcI may have a North/Central American origin [75–77]. It should be noted that if the current trend is to propose sub groups within TcI, the presence of subunits, evolving separately, must be previously evidenced which is not yet the case. Also, it has been proposed that marsupial species of the family Didelphidae family are the ancestral hosts of TcI [78] given that, among others, TcI predominates in these animals. Based on our recent analysis of *COII* and *CytB* gene sequences previously deposited in GenBank [8], we evaluated the haplotype and nucleotide diversities of TcI within the order Didelphimorphia, and we observed that these indices were comparable to those obtained for all the other orders of wild mammals combined. This assesses the larger genetic diversity in marsupials than in other animals, supporting a longer association. The remarkable expansion of TcI, which invaded most of environments, does not allow its origin to be determined from the picture of its geographical distribution alone.

TcII is a DTU as old as TcI, but it has been sampled much more rarely. The strains belonging to this DTU carry mitochondrial genes (mtTcII mitochondrial cluster) whose sequences show substantial genetic divergence from TcI. Moreover, this DTU presents a much lower genetic diversity than TcI. For example, the haplotype diversity of *COII* and *CytB* genes are 0.39 and 0.48, while for mtTcI they are 0.81 and 0.58 respectively [8]. A similar level of differences is also observed for nucleotide diversity. The available data on the geographical distribution of TcII suggest that it is absent or extremely rare in some ecosystems (Central and North America). It seems that TcII strains would not have had the same expansion capacity as TcI among the wild cycles, and they probably found refuge mostly in certain wild mammals. TcII is already reported in different wild mammals of the Chiroptera, Cingulate, Didelphimorphia and Primate orders. However, its strong association with primates in the Atlantic Coastal Rainforest in Brazil should be noted [79]. In humans, it is relatively abundant, accounting for 20% of human strains, but it is highly abundant in Brazil (66% of human strains identifications) and rare in most other countries except Colombia (15%) and Chile (30%). For now, its geographical distribution is more consistent with a South American origin, and further south than north of the Amazon basin where this DTU is more abundant.

TcIII and TcIV are DTUs that do not seem to be present throughout the entire endemic area. First, it is important to note that the genetic data do not clearly define these two groups separately. The genetic diversity of TcIII-TcIV is very large and the monophyly of each DTU is not really highlighted. Several studies showed that these strains are the result of ancient hybridization(s) between TcI and TcII strains, which suffer over time from genetic rearrangements, decreasing their level of heterozygosity at the expense of mosaic mitochondrial and nuclear genes [80]. Recombination events have probably occurred several times and this would have given a mtTcIII group composed of polyphyletic subgroups of strains. Therefore, the wild strains from the US, attributed to TcIV, seem to be a monophyletic subgroup differing from the others long ago [81], but whose closest ancestors have probably disappeared. There is little doubt that TcIII and TcIV DTUs have a sylvan origin, but these strains infect humans more than occasionally: the current database shows that TcIV is reported in 84 human cases in six countries (Brazil, Colombia, Ecuador, Guatemala, Peru and Venezuela), and 11 canine cases. Similarly, TcIII is reported in 26 human cases in Brazil and Paraguay.

The two TcV and TcVI DTUs include strains derived from the hybridization of TcII and TcIII strains [9]. They are usually considered hybrids and they are heterozygous at several loci and SNPs (single nucleotide polymorphisms). In our database, a total of 21.3% of the determinations belong to these DTUs. Some of these strains have spread across large geographic areas through the clonal propagation mode [82]. Both DTUs are clearly associated with domestic cycles since only 10.5% of them are identified in hosts from wild cycles. They are identified in some Didelphimorphia and in different species of rodents but only in the Southern Cone countries and Bolivia. Previously, the Gran Chaco region was proposed as the original location of these DTUs, where they are very abundant and where the putative parents are also present [15], and this hypothesis fits well with the current observed distribution of these DTUs.

### Host specificity

The universe of Hemiptera vectors of *T*. *cruzi* or potential vectors is huge since currently over 141–147 triatomine species are recognized, about 130 occur in the Americas, and it appears that all of these are able to transmit the parasite. Most of these species are involved in wild cycles with at least 100 species of mammals playing a role of host and/or reservoir. In the current data, only 37 species of vectors are included and for the majority of them, very few DTU determinations were made, even though these vectors are generally widely distributed. Similarly, the knowledge of the parasite genetic variants that infect mammals, except for humans, and to a lesser extent for Didelphidae, is very limited. In various regions, in a context of high anthropization and climate changes, it is urgent to study the impact of these environmental modifications on potential vectors and their hosts.

Several studies of experimental infections of vectors with different strains of *T*. *cruzi* showed differences in susceptibility [83] and even suggested that the strains are pathogenic and induce more or less deleterious effects in bugs [84]. Few studies relate comparisons of DTUs in experimental infections in a single triatomine species. For *T*. *infestans* in which this was done, significant developmental differences in the vector were observed depending on the DTU it was infected with, and after experimental double infections: in 50% of cases, only one of the two DTUS was detected after a few days of infection [85, 86]. As a field observation, we can report the case of *Triatoma sordida*, a primary vector in the northeast of the city of Santa Cruz, in Bolivia, in which TcI was predominantly detected while in mammals of the same area, TcV was a major strain [59]. In wild mammal hosts, experimental infections of two important reservoirs in the US (placental and marsupial) showed DTU-mammal association [87].

Examples could be multiplied but we can already conclude that the vectors and even the wild mammal hosts can influence the distribution of DTUs. Whatever the host, there is a balance between parasite genotypes and hosts which probably depends on environmental conditions such as outside temperature for vectors or immune and nutritional status for mammals. The diversity of hosts, and environmental conditions certainly explain the maintenance of parasitic diversity and the emergence of new variants by natural selection. Therefore the distribution of DTUs reported here, although very informative, is only a temporary picture that will inevitably evolve over time, above all if drastic environmental changes occur such as deforestation, intensive farming, urbanization, and unexpected climatic upheavals.

## Supporting Information

S1 Table Compiled list of *Trypanosoma cruzi* discrete typing units (DTU) previously reported in the literature. Each line corresponds to the identification of a single strain for which the geographic, host, and time origins are specified. The publication source is also presented.
(XLSX)

S2 Table Summary of the collected determinations of *T*. *cruzi* DTUs per region, country, hosts, and ecotope originating from the compilations of the data (S1 Table). (XLSX)

## References

[1] Tibayrenc M, Kjellberg F, Ayala FJ. A clonal theory of parasitic protozoa: the population structures of *Entamoeba*, *Giardia*, *Leishmania*, *Naegleria*, *Plasmodium*, *Trichomonas*, and *Trypanosoma* and their medical and taxonomical consequences. Proceedings of the National Academy of Sciences USA. 1990;87(7):2414–8. 10.1073/pnas.87.7.2414 PMC53699 2320563

[2] Ocana-Mayorga S, Llewellyn MS, Costales JA, Miles MA, Grijalva MJ. Sex, subdivision, and domestic dispersal of *Trypanosoma cruzi* lineage I in southern Ecuador. PLoS Negl Trop Dis. 2010;4(12):e915 10.1371/journal.pntd.0000915 21179502 PMC3001902

[3] Barnabé C, Buitrago R, Brémond P, Aliaga C, Salas R, Vidaurre P, et al Putative panmixia in restricted populations of *Trypanosoma cruzi* isolated from wild *Triatoma infestans* in Bolivia. PLoS One. 2013;8(11):e82269 10.1371/journal.pone.0082269 24312410 PMC3843716

[4] Messenger LA, Miles MA. Evidence and importance of genetic exchange among field populations of *Trypanosoma cruzi*. Acta Trop. 2015;151:150–5. 10.1016/j.actatropica.2015.05.007 26188331 PMC4644990

[5] Zingales B, Miles MA, Campbell DA, Tibayrenc M, Macedo AM, Teixeira MM, et al The revised *Trypanosoma cruzi* subspecific nomenclature: Rationale, epidemiological relevance and research applications. Infect Genet Evol. 2012;12(2):240–53. 10.1016/j.meegid.2011.12.009 22226704

[6] Zingales B, Andrade SG, Briones MR, Campbell DA, Chiari E, Fernandes O, et al A new consensus for *Trypanosoma cruzi* intraspecific nomenclature: second revision meeting recommends TcI to TcVI. Mem Inst Oswaldo Cruz. 2009;104(7):1051–4. 20027478 10.1590/s0074-02762009000700021

[7] Marcili A, Lima L, Cavazzana MJ, Junqueira ACV, Veludo HH, Da Silva FM, et al A new genotype of *Trypanosoma cruzi* associated with bats evidenced by phylogenetic analyses using SSU rDNA, cytochrome b and Histone H2B genes and genotyping based on ITS1 rDNA. Parasitology. 2009;136(6):641–55. 10.1017/S0031182009005861 19368741

[8] Barnabé C, Mobarec HI, Jurado MR, Cortez JA, Brenière SF. Reconsideration of the seven discrete typing units within the species, a new proposal of three reliable mitochondrial clades. Infect Genet Evol. 2016;39:176–86. 10.1016/j.meegid.2016.01.029 26845347

[9] Westenberger SJ, Barnabé C, Campbell DA, Sturm NR. Two hybridization events define the population structure of *Trypanosoma cruzi*. Genetics. 2005;171(2):527–43. 15998728 10.1534/genetics.104.038745 PMC1456769

[10] Ramirez JD, Duque MC, Guhl F. Phylogenetic reconstruction based on Cytochrome b (Cytb) gene sequences reveals distinct genotypes within Colombian I populations. Acta Trop. 2011;119(1):61–5. 10.1016/j.actatropica.2011.04.009 21524641

[11] Sturm NR, Vargas NS, Westenberger SJ, Zingales B, Campbell DA. Evidence for multiple hybrid groups in *Trypanosoma cruzi*. Int J Parasitol. 2003;33(3):269–79. 12670512 10.1016/s0020-7519(02)00264-3

[12] Tomasini N, Diosque P. Evolution of: clarifying hybridisations, mitochondrial introgressions and phylogenetic relationships between major lineages. Mem Inst Oswaldo Cruz. 2015;110(3):403–13. 10.1590/0074-02760140401 25807469 PMC4489478

[13] de Freitas JM, Augusto-Pinto L, Pimenta JR, Bastos-Rodrigues L, Goncalves VF, Teixeira SM, et al Ancestral Genomes, Sex, and the Population Structure of. PLoS Pathog. 2006;2(3):e24 16609729 10.1371/journal.ppat.0020024 PMC1434789

[14] Brenière SF, Aliaga C, Waleckx E, Buitrago R, Salas R, Barnabé C, et al Genetic characterization of DTUs in wild *Triatoma infestans* from Bolivia: predominance of TcI. PLoS Negl Trop Dis. 2012;6(5):e1650 10.1371/journal.pntd.0001650 22685616 PMC3368956

[15] Perez E, Monje M, Chang B, Buitrago R, Parrado R, Barnabé C, et al Predominance of hybrid discrete typing units of in domestic *Triatoma infestans* from the Bolivian Gran Chaco region. Infect Genet Evol. 2013;13:116–23. 10.1016/j.meegid.2012.09.014 23047136

[16] Cavazzana M Jr., Marcili A, Lima L, da Silva FM, Junqueira AC, Veludo HH, et al Phylogeographical, ecological and biological patterns shown by nuclear (ssrRNA and gGAPDH) and mitochondrial (Cyt b) genes of trypanosomes of the subgenus Schizotrypanum parasitic in Brazilian bats. Int J Parasitol. 2010;40(3):345–55. 10.1016/j.ijpara.2009.08.015 19766649

[17] Ramirez JD, Tapia-Calle G, Munoz-Cruz G, Poveda C, Rendon LM, Hincapie E, et al Trypanosome species in neo-tropical bats: biological, evolutionary and epidemiological implications. Infect Genet Evol. 2014;22:250–6. 10.1016/j.meegid.2013.06.022 23831017 PMC7106241

[18] Ramirez JD, Hernandez C, Montilla M, Zambrano P, Florez AC, Parra E, et al First report of human infection attributed to TcBat genotype. Zoonoses Public Health. 2014;61(7):477–9. 10.1111/zph.12094 25285940

[19] Brenière SF, Bosseno MF, Telleria J, Bastrenta B, Yacsik N, Noireau F, et al Different behavior of two major clones: transmission and circulation in young Bolivian patients. Exp Parasitol. 1998;89(3):285–95. 9676706 10.1006/expr.1998.4295

[20] Devillers H, Lobry JR, Menu F. An agent-based model for predicting the prevalence of I and II in their host and vector populations. J Theor Biol. 2008;255(3):307–15. 10.1016/j.jtbi.2008.08.023 18805428

[21] Bosseno MFY, Vargas N., Brenière F., S. F. Selection of clonal genotypes (clonet 20 and 39) isolated from Bolivian triatomines following subculture in liquid medium. Memorias Do Instituto Oswaldo Cruz. 2000;95(5):601–7. 10998206 10.1590/s0074-02762000000500002

[22] Tibayrenc M, Barnabé C, Telleria J. Reticulate Evolution in: Medical and Epidemiological Implications In: Telleria J, Tibayrenc M, editors. American trypanosomiasis: Chagas disease One hundred years of research. Burlington: Elsevier; 2010 p. 475–88.

[23] Miles MA, Llewellyn MS, Lewis MD, Yeo M, Baleela R, Fitzpatrick S, et al The molecular epidemiology and phylogeography of and parallel research on Leishmania: looking back and to the future. Parasitology. 2009;136(12):1509–28. 10.1017/S0031182009990977 19691868

[24] Barnabé C, Brisse S, Tibayrenc M. Population structure and genetic typing of, the agent of Chagas disease: a multilocus enzyme electrophoresis approach. Parasitology. 2000;120:513–26. 10840981 10.1017/s0031182099005661

[25] Brisse S, Verhoef J, Tibayrenc M. Characterisation of large and small subunit rRNA and mini-exon genes further supports the distinction of six *Trypanosoma cruzi* lineages. Int J Parasitol. 2001;31(11):1218–26. 11513891 10.1016/s0020-7519(01)00238-7

[26] Lima L, Espinosa-Alvarez O, Pinto CM, Cavazzana M Jr., Pavan AC, Carranza JC, et al New insights into the evolution of the *Trypanosoma cruzi* clade provided by a new trypanosome species tightly linked to Neotropical Pteronotus bats and related to an Australian lineage of trypanosomes. Parasit Vectors. 2015;8:657 10.1186/s13071-015-1255-x 26701154 PMC4690318

[27] Pinto CM, Ocana-Mayorga S, Tapia EE, Lobos SE, Zurita AP, Aguirre-Villacis F, et al Bats, Trypanosomes, and Triatomines in Ecuador: New Insights into the Diversity, Transmission, and Origins of *Trypanosoma cruzi* and Chagas Disease. PLoS One. 2015;10(10):e0139999 10.1371/journal.pone.0139999 26465748 PMC4605636

[28] Cominetti MC, Csordas BG, Cunha RC, Andreotti R. Geographical distribution of *Trypanosoma cruzi* in triatomine vectors in the State of Mato Grosso do Sul, Brazil. Rev Soc Bras Med Trop. 2014;47(6):747–55. 10.1590/0037-8682-0234-2014 25626654

[29] Ramos-Ligonio A, Torres-Montero J, Lopez-Monteon A, Dumonteil E. Extensive diversity of *Trypanosoma cruzi* discrete typing units circulating in *Triatoma dimidiata* from central Veracruz, Mexico. Infect Genet Evol. 2012;12(7):1341–3. 10.1016/j.meegid.2012.04.024 22569098

[30] Ibanez-Cervantes G, Martinez-Ibarra A, Nogueda-Torres B, Lopez-Orduna E, Alonso AL, Perea C, et al Identification by Q-PCR of *Trypanosoma cruzi* lineage and determination of blood meal sources in triatomine gut samples in Mexico. Parasitol Int. 2013;62(1):36–43. 10.1016/j.parint.2012.09.003 22995149

[31] Herrera CP, Licon MH, Nation CS, Jameson SB, Wesson DM. Genotype diversity of *Trypanosoma cruzi* in small rodents and Triatoma sanguisuga from a rural area in New Orleans, Louisiana. Parasit Vectors. 2015;8:123 10.1186/s13071-015-0730-8 25890064 PMC4344744

[32] Dorn PL, Perniciaro L, Yabsley MJ, Roellig DM, Balsamo G, Diaz J, et al Autochthonous transmission of *Trypanosoma cruzi*, Louisiana. Emerg Infect Dis. 2007;13(4):605–7. 17553277 10.3201/eid1304.061002 PMC2725963

[33] Bosseno MF, Barnabé C, Magallon Gastelum E, Lozano Kasten F, Ramsey J, Espinoza B, et al Predominance of *Trypanosoma cruzi* Lineage I in Mexico. J Clin Microbiol. 2002;40(2):627–32. 11825982 10.1128/JCM.40.2.627-632.2002 PMC153397

[34] Bosseno MF, Barnabé C, Sierra MJ, Kengne P, Guerrero S, Lozano F, et al Wild ecotopes and food habits of *Triatoma longipennis* infected by *Trypanosoma cruzi* lineages I and II in Mexico. Am J Trop Med Hyg. 2009;80(6):988–91. 19478263

[35] Roellig DM, Savage MY, Fujita AW, Barnabé C, Tibayrenc M, Steurer FJ, et al Genetic variation and exchange in *Trypanosoma cruzi* isolates from the United States. PLoS One. 2013;8(2):e56198 10.1371/journal.pone.0056198 23457528 PMC3572986

[36] Shender LA, Lewis MD, Rejmanek D, Mazet JA. Molecular Diversity of *Trypanosoma cruzi* Detected in the Vector *Triatoma protracta* from California, USA. PLoS Negl Trop Dis. 2016;10(1):e0004291 10.1371/journal.pntd.0004291 26797311 PMC4721664

[37] Iwagami M, Higo H, Miura S, Yanagi T, Tada I, Kano S, et al Molecular phylogeny of *Trypanosoma cruzi* from Central America (Guatemala) and a comparison with South American strains. Parasitol Res. 2007;102(1):129–34. 17828552 10.1007/s00436-007-0739-9

[38] Higo H, Miura S, Horio M, Mimori T, Hamano S, Agatsuma T, et al Genotypic variation among lineages of *Trypanosoma cruzi* and its geographic aspects. Parasitol Int. 2004;53(4):337–44. 15464443 10.1016/j.parint.2004.06.001

[39] Brisse S, Henriksson J, Barnabé C, Douzery EJ, Berkvens D, Serrano M, et al Evidence for genetic exchange and hybridization in *Trypanosoma cruzi* based on nucleotide sequences and molecular karyotype. Infect Genet Evol. 2003;2(3):173–83. 12797979 10.1016/s1567-1348(02)00097-7

[40] Ramirez JD, Turriago B, Tapia-Calle G, Guhl F. Understanding the role of dogs (Canis lupus familiaris) in the transmission dynamics of *Trypanosoma cruzi* genotypes in Colombia. Vet Parasitol. 2013;196(1–2):216–9. 10.1016/j.vetpar.2012.12.054 23351975

[41] Sanchez LV, Bautista DC, Corredor AF, Herrera VM, Martinez LX, Villar JC, et al Temporal variation of *Trypanosoma cruzi* discrete typing units in asymptomatic Chagas disease patients. Microbes Infect. 2013;15(10–11):745–8. 10.1016/j.micinf.2013.06.008 23811021

[42] Garzon EA, Barnabé C, Cordova X, Bowen C, Paredes W, Gomez E, et al *Trypanosoma cruzi* isoenzyme variability in Ecuador: first observation of zymodeme III genotypes in chronic chagasic patients. Trans R Soc Trop Med Hyg. 2002;96(4):378–82. 12497973 10.1016/s0035-9203(02)90367-6

[43] Brenière SF, Braquemond P, Solari A, Agnèse JF, Tibayrenc M. An isoenzyme study of naturally occurring clones of *Trypanosoma cruzi* isolated from both sides of the West Andes highland. Transactions of the Royal Society of Tropical Medicine and Hygiene. 1991;85(1):62–6. 2068764 10.1016/0035-9203(91)90160-z

[44] Guhl F, Auderheide A, Ramirez JD. From ancient to contemporary molecular eco-epidemiology of Chagas disease in the Americas. Int J Parasitol. 2014;44(9):605–12. 10.1016/j.ijpara.2014.02.005 24675555

[45] Walter A, Lozano-Kasten F, Bosseno MF, Ruvalcaba EG, Gutierrez MS, Luna CE, et al Peridomicilary habitat and risk factors for *Triatoma* infestation in a rural community of the Mexican occident. Am J Trop Med Hyg. 2007;76(3):508–15. 17360876

[46] Rojas-Cortez M, Pinazo MJ, Garcia L, Arteaga M, Uriona L, Gamboa S, et al *Trypanosoma cruzi*-infected *Panstrongylus geniculatus* and *Rhodnius robustus* adults invade households in the Tropics of Cochabamba region of Bolivia. Parasit Vectors. 2016;9(1):158. 26984679 10.1186/s13071-016-1445-1 PMC4794895

[47] Carrasco HJ, Segovia M, Llewellyn MS, Morocoima A, Urdaneta-Morales S, Martinez C, et al Geographical distribution of *Trypanosoma cruzi* genotypes in Venezuela. PLoS Negl Trop Dis. 2012;6(6):e1707 10.1371/journal.pntd.0001707 22745843 PMC3383755

[48] Ferreira RC, Briones MR. Phylogenetic evidence based on *Trypanosoma cruzi* nuclear gene sequences and information entropy suggest that inter-strain intragenic recombination is a basic mechanism underlying the allele diversity of hybrid strains. Infect Genet Evol. 2012;12(5):1064–71. 10.1016/j.meegid.2012.03.010 22449773

[49] Lima Vdos S, Xavier SC, Maldonado IF, Roque AL, Vicente AC, Jansen AM. Expanding the knowledge of the geographic distribution of *Trypanosoma cruzi* TcII and TcV/TcVI genotypes in the Brazilian Amazon. PLoS One. 2014;9(12):e116137 10.1371/journal.pone.0116137 25551227 PMC4281250

[50] Valadares HM, Pimenta JR, de Freitas JM, Duffy T, Bartholomeu DC, Oliveira Rde P, et al Genetic profiling of *Trypanosoma cruzi* directly in infected tissues using nested PCR of polymorphic microsatellites. Int J Parasitol. 2008;38(7):839–50. 18154957 10.1016/j.ijpara.2007.10.017

[51] Arenas M, Campos R, Coronado X, Ortiz S, Solari A. *Trypanosoma cruzi* genotypes of insect vectors and patients with Chagas of Chile studied by means of cytochrome b gene sequencing, minicircle hybridization, and nuclear gene polymorphisms. Vector Borne Zoonotic Dis. 2012;12(3):196–205. 10.1089/vbz.2011.0683 22022808 PMC3300063

[52] Coronado X, Rozas M, Botto-Mahan C, Ortiz S, Cattan PE, Solari A. Molecular epidemiology of Chagas disease in the wild transmission cycle: the evaluation in the sylvatic vector *Mepraia spinolai* from an endemic area of Chile. Am J Trop Med Hyg. 2009;81(4):656–9. 10.4269/ajtmh.2009.09-0053 19815882

[53] Toledo A, Vergara F, Campos R, Botto-Mahan C, Ortiz S, Coronado X, et al *Trypanosoma cruzi* genotypes in Mepraia gajardoi from wild ecotopes in northern Chile. Am J Trop Med Hyg. 2013;88(2):285–8. 10.4269/ajtmh.2012.12-0227 23249691 PMC3583318

[54] Abolis NG, Araujo SM, Toledo MJ, Fernandez MA, Gomes ML. *Trypanosoma cruzi* I-III in southern Brazil causing individual and mixed infections in humans, sylvatic reservoirs and triatomines. Acta Trop. 2011;120(3):167–72. 10.1016/j.actatropica.2011.08.001 21855523

[55] Maffey L, Cardinal MV, Ordonez-Krasnowski PC, Lanati LA, Lauricella MA, Schijman AG, et al Direct molecular identification of *Trypanosoma cruzi* discrete typing units in domestic and peridomestic *Triatoma infestans* and *Triatoma sordida* from the Argentine Chaco. Parasitology. 2012;139(12):1570–9. 10.1017/S0031182012000856 23036510 PMC3749237

[56] Cardinal MV, Lauricella MA, Ceballos LA, Lanati L, Marcet PL, Levin MJ, et al Molecular epidemiology of domestic and sylvatic *Trypanosoma cruzi* infection in rural northwestern Argentina. Int J Parasitol. 2008. 10.1016/j.ijpara.2008.04.010 PMC3143243 18585717

[57] Enriquez GF, Cardinal MV, Orozco MM, Lanati L, Schijman AG, Gurtler RE. Discrete typing units of *Trypanosoma cruzi* identified in rural dogs and cats in the humid Argentinean Chaco. Parasitology. 2013;140(3):303–8. 10.1017/S003118201200159X 23058180 PMC3721149

[58] Rozas M, Botto-Mahan C, Coronado X, Ortiz S, Cattan PE, Solari A. Coexistence of *Trypanosoma cruzi* genotypes in wild and periodomestic mammals in Chile. Am J Trop Med Hyg. 2007;77(4):647–53. 17978065

[59] Brenière SF, Morochi W, Bosseno MF, Ordonez J, Gutierrez T, Vargas F, et al *Trypanosoma cruzi* genotypes associated with domestic *Triatoma sordida* in Bolivia. Acta Trop. 1998;71(3):269–83. 9879736 10.1016/s0001-706x(98)00061-8

[60] Herrera HM, Rocha FL, Lisboa CV, Rademaker V, Mourao GM, Jansen AM. Food web connections and the transmission cycles of *Trypanosoma cruzi* and *Trypanosoma evansi* (Kinetoplastida, Trypanosomatidae) in the Pantanal Region, Brazil. Trans R Soc Trop Med Hyg. 2011;105(7):380–7. 10.1016/j.trstmh.2011.04.008 21600622

[61] De Araujo VA, Boite MC, Cupolillo E, Jansen AM, Roque AL. Mixed infection in the anteater Tamandua tetradactyla (Mammalia: Pilosa) from Para State, Brazil: *Trypanosoma cruzi*, *T*. *rangeli* and *Leishmania infantum*. Parasitology. 2013;140(4):455–60. 10.1017/S0031182012001886 23253893

[62] Roque AL, Xavier SC, Gerhardt M, Silva MF, Lima VS, D'Andrea PS, et al *Trypanosoma cruzi* among wild and domestic mammals in different areas of the Abaetetuba municipality (Para State, Brazil), an endemic Chagas disease transmission area. Vet Parasitol. 2013;193(1–3):71–7. 10.1016/j.vetpar.2012.11.028 23261089

[63] Lisboa CV, Xavier SC, Herrera HM, Jansen AM. The ecology of the *Trypanosoma cruzi* transmission cycle: Dispersion of zymodeme 3 (Z3) in wild hosts from Brazilian biomes. Vet Parasitol. 2009. 10.1016/j.vetpar.2009.07.002 19643545

[64] Marcili A, Lima L, Valente VC, Valente SA, Batista JS, Junqueira AC, et al Comparative phylogeography of *Trypanosoma cruzi* TCIIc: New hosts, association with terrestrial ecotopes, and spatial clustering. Infect Genet Evol. 2009. 10.1016/j.meegid.2009.07.003 19632356

[65] Llewellyn MS, Lewis MD, Acosta N, Yeo M, Carrasco HJ, Segovia M, et al *Trypanosoma cruzi* IIc: Phylogenetic and Phylogeographic Insights from Sequence and Microsatellite Analysis and Potential Impact on Emergent Chagas Disease. PLoS Negl Trop Dis. 2009;3(9):e510 10.1371/journal.pntd.0000510 19721699 PMC2727949

[66] Fernandes O, Souto RP, Castro JA, Pereira JB, Fernandes NC, Junqueira AC, et al Brazilian isolates of *Trypanosoma cruzi* from humans and triatomines classified into two lineages using mini-exon and ribosomal RNA sequences. Am J Trop Med Hyg. 1998;58(6):807–11. 9660469 10.4269/ajtmh.1998.58.807

[67] Lopez-Cancino SA, Tun-Ku E, De la Cruz-Felix HK, Ibarra-Cerdena CN, Izeta-Alberdi A, Pech-May A, et al Landscape ecology of *Trypanosoma cruzi* in the southern Yucatan Peninsula. Acta Trop. 2015;151:58–72. 10.1016/j.actatropica.2015.07.021 26219998

[68] Barnabé C, De Meeus T, Noireau F, Bosseno MF, Monje EM, Renaud F, et al *Trypanosoma cruzi* discrete typing units (DTUs): microsatellite loci and population genetics of DTUs TcV and TcI in Bolivia and Peru. Infect Genet Evol. 2011;11(7):1752–60. 10.1016/j.meegid.2011.07.011 21801854

[69] Yeo M, Mauricio IL, Messenger LA, Lewis MD, Llewellyn MS, Acosta N, et al Multilocus sequence typing (MLST) for lineage assignment and high resolution diversity studies in *Trypanosoma cruzi*. PLoS Negl Trop Dis. 2011;5(6):e1049 10.1371/journal.pntd.0001049 21713026 PMC3119646

[70] Lauthier JJ, Tomasini N, Barnabé C, Rumi MM, D'Amato AM, Ragone PG, et al Candidate targets for Multilocus Sequence Typing of *Trypanosoma cruzi*: Validation using parasite stocks from the Chaco Region and a set of reference strains. Infect Genet Evol. 2012. 10.1016/j.meegid.2011.12.008 22210092

[71] Lewis MD, Ma J, Yeo M, Carrasco HJ, Llewellyn MS, Miles MA. Genotyping of *Trypanosoma cruzi*: systematic selection of assays allowing rapid and accurate discrimination of all known lineages. Am J Trop Med Hyg. 2009;81(6):1041–9. 10.4269/ajtmh.2009.09-0305 19996435 PMC2825677

[72] Carrasco HJ, Frame IA, Valente SA, Miles MA. Genetic exchange as a possible source of genomic diversity in sylvatic populations of *Trypanosoma cruzi*. Am J Trop Med Hyg. 1996;54(4):418–24. 8615458 10.4269/ajtmh.1996.54.418

[73] O'Connor O, Bosseno MF, Barnabé C, Douzery EJ, Brenière SF. Genetic clustering of *Trypanosoma cruzi* I lineage evidenced by intergenic miniexon gene sequencing. Infect Genet Evol. 2007;7(5):587–93. 17553755 10.1016/j.meegid.2007.05.003

[74] Cura CI, Mejia-Jaramillo AM, Duffy T, Burgos JM, Rodriguero M, Cardinal MV, et al *Trypanosoma cruzi* I genotypes in different geographical regions and transmission cycles based on a microsatellite motif of the intergenic spacer of spliced-leader genes. Int J Parasitol. 2010;40:1599–607. 10.1016/j.ijpara.2010.06.006 20670628 PMC3081674

[75] Herrera CP, Barnabé C, Brenière SF. Complex evolutionary pathways of the intergenic region of the mini-exon gene in *Trypanosoma cruzi* TcI: A possible ancient origin in the Gran Chaco and lack of strict genetic structuration. Infect Genet Evol. 2013;16:27–37. 10.1016/j.meegid.2012.12.028 23380053

[76] Llewellyn MS, Miles MA, Carrasco HJ, Lewis MD, Yeo M, Vargas J, et al Genome-scale multilocus microsatellite typing of *Trypanosoma cruzi* discrete typing unit I reveals phylogeographic structure and specific genotypes linked to human infection. PLoS Pathog. 2009;5(5):e1000410 10.1371/journal.ppat.1000410 19412340 PMC2669174

[77] Ramirez JD, Guhl F, Messenger LA, Lewis MD, Montilla M, Cucunuba Z, et al Contemporary cryptic sexuality in *Trypanosoma cruzi*. Mol Ecol. 2012;21(17):4216–26. 10.1111/j.1365-294X.2012.05699.x 22774844

[78] Yeo M, Acosta N, Llewellyn M, Sanchez H, Adamson S, Miles GA, et al Origins of Chagas disease: Didelphis species are natural hosts of *Trypanosoma cruzi* I and armadillos hosts of Trypanosoma cruzi II, including hybrids. Int J Parasitol. 2005;35(2):225–33. 15710443 10.1016/j.ijpara.2004.10.024

[79] Lisboa CV, Monteiro RV, Martins AF, Xavier SC, Lima Vdos S, Jansen AM. Infection with *Trypanosoma cruzi* TcII and TcI in free-ranging population of lion tamarins (Leontopithecus spp): an 11-year follow-up. Mem Inst Oswaldo Cruz. 2015;110(3):394–402. 10.1590/0074-02760140400 25946156 PMC4489477

[80] Lewis MD, Llewellyn MS, Yeo M, Acosta N, Gaunt MW, Miles MA. Recent, independent and anthropogenic origins of *Trypanosoma cruzi* hybrids. PLoS Negl Trop Dis. 2011;5(10):e1363 10.1371/journal.pntd.0001363 22022633 PMC3191134

[81] Barnabé C, Yaeger R, Pung O, Tibayrenc M. *Trypanosoma cruzi*: a considerable phylogenetic divergence indicates that the agent of Chagas disease is indigenous to the native fauna of the United States. Exp Parasitol. 2001;99(2):73–9. 11748960 10.1006/expr.2001.4651

[82] Tibayrenc M, Ayala FJ. Isozyme variability in *Trypanosoma cruzi*, the agent of Chagas’ disease: genetical, taxonomical, and epidemiological significance. Evolution. 1988;42(2):277–92. 28567853 10.1111/j.1558-5646.1988.tb04132.x

[83] Campos R, Acuna-Retamar M, Botto-Mahan C, Ortiz S, Cattan PE, Solari A. Susceptibility of *Mepraia spinolai* and *Triatoma infestans* to different *Trypanosoma cruzi* strains from naturally infected rodent hosts. Acta Trop. 2007;104(1):25–9. 17904090 10.1016/j.actatropica.2007.07.005

[84] Peterson JK, Graham AL, Dobson AP, Chavez OT. *Rhodnius prolixus* life history outcomes differ when infected with different *Trypanosoma cruzi* I strains. Am J Trop Med Hyg. 2015. 10.4269/ajtmh.15-0218 PMC4559699 26078316

[85] Pinto AS, de Lana M, Bastrenta B, Barnabé C, Quesney V, Noel S, et al Compared vectorial transmissibility of pure and mixed clonal genotypes of *Trypanosoma cruzi* in *Triatoma infestans*. Parasitol Res. 1998;84(5):348–53. 9610630 10.1007/s004360050409

[86] de Lana M, da Silveira Pinto A, Barnabé C, Quesney V, Noël S, Tibayrenc M. *Trypanosoma cruzi*: compared vectorial transmissibility of three major clonal genotypes by *Triatoma infestans*. Exp Parasitol. 1998;90(1):20–5. 9709026 10.1006/expr.1998.4304

[87] Roellig DM, McMillan K, Ellis AE, Vandeberg JL, Champagne DE, Yabsley MJ. Experimental infection of two South American reservoirs with four distinct strains of *Trypanosoma cruzi*. Parasitology. 2010;137(6):959–66. 10.1017/S0031182009991995 20128943 PMC2915445

